# Phylogenetic Analysis Reveals Distinct Evolutionary Trajectories of the Fluoroquinolones-Resistant *Escherichia coli* ST1193 From Fuzhou, China

**DOI:** 10.3389/fmicb.2021.746995

**Published:** 2021-11-05

**Authors:** Jiangqing Huang, Zhichang Zhao, Qianwen Zhang, Shengcen Zhang, Shuyu Zhang, Min Chen, Hongqiang Qiu, Yingping Cao, Bin Li

**Affiliations:** ^1^Department of Clinical Laboratory, Fujian Medical University Union Hospital, Fuzhou, China; ^2^Department of Pharmacy, Fujian Medical University Union Hospital, Fuzhou, China; ^3^Department of Laboratory Medicine, Fujian Medical University, Fuzhou, China

**Keywords:** *E. coli*, ST1193, whole genome sequencing, phylogenomic analysis, *bla*_*CTX–M–55*_, fluoroquinolones-resistant

## Abstract

*Escherichia coli* (*E. coli*) ST1193 is an emerging fluoroquinolones-resistant and virulent lineage. Large gaps remain in our understanding of the evolutionary processes and differences of this lineage. Therefore, we used 76 *E. coli* ST1193 genomes to detect strain-level genetic diversity and phylogeny of this lineage globally. All *E. coli* ST1193 possessed *fimH64*, *filCH5*, and *fumC14*. There was 94.7% of isolates classified as O-type O75. There was 9.33% of *E. coli* ST1193 that possessed K5 capsular, while 90.67% of isolates possessed K1 capsular. The core genome analysis revealed that all isolates were divided into two phylogenetic clades (clade A and B). Clade A included 25 non-Chinese *E. coli* ST1193, and clade B contained all isolates collected from Fuzhou, China, respectively. The results of comparative genomics indicated Indels were identified in 150 clade-specific genes, which were enriched into the biological process and molecular function. Accessory genome phylogenetic tree showed a high degree of correlation between accessory genome clusters and core genome clades. There was significant difference in antibiotic resistance genes (ARGs) [*bla*_*CTX–M–55*_, *bla*_*TEM–1*_, *sul2*, *tet(B)*, *tet(R)*, *APH(6)-Id*, and *AAC(3)-IId*], virulence factors (*cia*, *neuC*, *gad*, and *traT)*, and plasmid replicon types (IncQ1, Col156, and IncB/O/K/Z) between clade A (non-Chinese isolates) and clade B (Chinese isolates) (*p* < 0.05). Further analysis of the genetic environments of *bla*_*CTX–M–55*_ demonstrated that the flanking contexts of *bla*_*CTX–M–55*_ were diverse. In conclusion, our results reveal the distinct evolutionary trajectories of the spread of *E. coli* ST1193 in Fuzhou, China and non-China regions. This supports both global transmission and localized lineage expansion of this lineage following specific introductions into a geographic locality.

## Introduction

*Escherichia coli* (*E. coli*) is the most common gram-negative bacterial opportunistic pathogen. It primarily inhabits the lower intestinal tract of humans and other warm-blooded animals, presenting both a clinical and an epidemiological challenge (Vila et al., 2016). *E. coli* could cause many clinical infections, ranging from diarrhea, uncomplicated urinary tract infections to life-threatening septicemia, leading to more than two million deaths each year worldwide (Gomes et al., 2016; Tandogdu and Wagenlehner, 2016; Micenková et al., 2017; Askari and Ghanbarpour, 2019).

Multi-locus sequence typing (MLST) is a convenient sequence-based method for identifying clonal relationships among bacteria and is usually applied to detect the population structure of *E. coli* strains with respect to their specificity for infecting humans or animals (Chaudhuri and Henderson, 2012; Riley, 2014). In the past 20 years, resistance to fluoroquinolones and extended-spectrum cephalosporins rapidly increased due to the production of extended-spectrumβ-lactamases by *E. coli* (Nicolas-Chanoine et al., 2014). This resistance increase is linked to the worldwide spread of a specific clone of *E. coli*, sequence type (ST) 131 (Nicolas-Chanoine et al., 2014). Previous studies demonstrated that *E. coli* ST131 accounted for nearly half in fluoroquinolones-resistant *E. coli* (Tchesnokova V. et al., 2019; Tchesnokova V. L. et al., 2019. Interestingly, the *E. coli* population structure is dynamic (Johnson T. J. et al., 2019). A new sequence type, *E. coli* ST1193, was reported as an emerging fluoroquinolones-resistant (FQ-R) and virulent *E. coli* lineage that usually causes various extraintestinal infections, such as septicemia, urinary tract infection, and meningitis (Wu et al., 2017; Johnson J. R. et al., 2019; Ding et al., 2021). This lineage was usually isolated from animals and humans in many countries, including the United States, China, and Australia (Platell et al., 2012; Wu et al., 2017; Xia et al., 2017; Johnson T. J. et al., 2019). Moreover, the percentage of this lineage rose dramatically from 4.4 to 22.2% during 2010–2017 and the growing frequency of this linage soared fivefold over the ST131 linage (Tchesnokova V. et al., 2019). Until now, *E. coli* ST1193 became a sizable second clonal group behind ST131-H30. Therefore, the increase and rapid dissemination of *E. coli* ST1193 are of great concern.

Most of the studies recently focused on the epidemiological investigation among *E. coli* ST1193. A previous study illustrated probable key events leading to *E. coli* ST1193 lineage, but it could not verify geographical isolation sources and gain a solid conclusion regarding geographical clustering (Johnson T. J. et al., 2019). Information on geographical clustering in Chinese ST1193 strains is limited. Large gaps remain in our understanding regarding the evolutionary processes and differences of this lineage. This study aimed to detect strain-level genetic diversity and phylogeny of *E. coli* ST1193 lineage using whole genome sequencing and gain a deep insight into this lineage globally, and to investigate the presence of genetic elements related to antimicrobial resistance (AMR) that might confer selective advantages to this emerging bacterial pathogen.

## Materials and Methods

### Bacterial Isolates and Whole Genome Sequencing

A total of 76 *E. coli* ST1193 were used in this study. Among them, 51 isolates were collected at Fujian Medical University Union Hospital (Fuzhou, China) between August 2014 and August 2015, reported in our previous study (Wu et al., 2017). These 51 *E. coli* ST1193 isolates were subjected to whole genome sequencing (WGS). Isolates were grown overnight from a single colony in 2 ml LB broth. The genomic DNA samples were extracted from *E. coli* ST1193 isolates by TIANamp Bacteria DNA Kit (Tiangen, Beijing) according to the manufacturer’s instructions. They were sequenced using the Illumina NovaSeq platform at Shanghai Personal Biotechnology Co., Ltd. (Shanghai, China). Then, FastQC software (version.0.11.7)^1^ was used to detect the quality of raw data and data assembly was proceeding after adapter contamination removing and data filtering using AdapterRemoval (version.2.2.2) (Lindgreen, 2012) and SOAPec (version.2.03) (Luo et al., 2012) based on Kmer frequency. The filtered reads were assembled by SPAdes (version. 3.12.0) (Bankevich et al., 2012), and A5-miseq (version 20160825) (Coil et al., 2015) to constructed scaffolds and contigs. Pilon software (version 1.18) (Walker et al., 2014) was used for base correction. Genome annotation for each assembly was performed using Prokka (version.1.12) (Seemann, 2014) with *E. coli* serving as the target species. Those 51 *E. coli* ST1193 genomes were submitted to GenBank under Bioproject PRJNA670319 and released on January 2, 2021.

Enterobase was a repository for *E. coli* genomic data^2^, and was also searched for isolates belonging to ST1193 (according to the Achtman 7-gene MLST scheme, including *adk*, *fumC*, *gyrB*, *icd*, *mdh*, *purA* and *recA*) with accession numbers linked to the National Center for Biotechnology Information (NCBI) database^3^ (Zhou et al., 2020). The remaining 25 *E. coli* ST1193 were obtained from NCBI. A total of 25 assembled *E. coli* ST1193 genomes were released on NCBI on April 1, 2020, which were included in this study for further analysis. The Accession numbers for these 25 isolates are listed in Supplementary Table 1. Moreover, the metadata information (strain name, source, isolated date, and country of origin) was collected and coupled with the phylogenetic tree for further analysis. All 25 *E. coli* ST1193 genomes were provided with new annotation using Prokka.

### Genome Data Analysis

The Center for Genomic Epidemiology tools^4^ were used to identify the gene contents. Specifically, plasmid types were identified using PlasmidFinder (version 2.0.1) and pMLST (version 0.1.0) (95% minimum match and 60% minimum length); virulence factors (VFs) were detected using VirulenceFinder (version 2.0.3) (90% minimum match and 60% minimum length); the multilocus-sequence typing was analyzed using MLST (version 2.0.4); serotypes and *fimH* alleles were predicted with SerotypeFinder (version 2.0.1) (85% minimum match and 60% minimum length) and FimTyper (version 1.0) (95% minimum match), respectively.

In addition, CARD (version 3.1.3)^5^ was used to determine resistance genes and known chromosomal mutations conferring antibiotic resistance (Alcock et al., 2020). CRISPRcasFinder^6^ (Couvin et al., 2018) was used with default parameters to identify the CRISPR loci in the genomes and determine the number and sequences of the spacers within CRISPR repeat arrays. The only CRISPR with evidence-levels 3 and 4 were considered as highly likely CRISPR arrays in this study according to the instructions (Couvin et al., 2018).

### Core Genome Analysis and Accessory Genome Analysis

First, the sequence was classified into gene families. The potential homologous genes were detected by sequence similarity and all homologous genes in each *E. coli* ST1193 genome were clustered into a single gene family by OrthoFinder software (version 2.3.12) (Emms and Kelly, 2015). The BlastP tool (Li and Lu, 2019) was used to compare the protein sequences from all genomes with an e-value cutoff set at 10^–10^ and an additional matching length criterion set at 85% of the query sequence. In order to further analyze the gene family, the mafft (version 7.429) software (Katoh et al., 2002) was applied to compare the sequence of gene families. Meanwhile, the sequences were annotated using clusters of orthologous groups of proteins (COGs) database by BlastP with an e-value cutoff of 10^–10^ as described previously (Li et al., 2017).

Core genomes were defined as described previously (McNally et al., 2016). Based on the comparative analysis of 76 *E. coli* ST1193, core genomes were selected as single-copy core-genome for sequence alignment using mafft (Cazares et al., 2020). The set of single-copy core-genome clusters detected was entered into the pipeline to identify high-quality phylogenetic markers and infer a core genome phylogeny through a maximum-likelihood tree search. Following extraction of core genome sequences, the remaining sequences of 76 *E. coli* ST1193 were designated as accessory genomes (Ozer et al., 2014). The data set of accessory genomes was composed of single-copy genes shared by 50% or more genomes, but less than 100% genomes and those accessory genomes were selected for sequence alignment using mafft. Maximum likelihood phylogeny of core and accessory genome of 76 *E. coli* were inferred from the alignment using FastTree (version 2.1.11) with Whelan-and-Goldman 2001 model and 1,000 bootstrap replicates, respectively (Price et al., 2009). The resulting phylogenetic tree was visualized using iTOL (version 6) which was also used to overlay metadata information (Letunic and Bork, 2019). Furthermore, the results of clusters of accessory genome phylogenetic tree were visualized in combination with the corresponding core genome phylogenetic tree using iTOL.

### Gene Ontology Term Enrichment Analysis

The SNP and indels of differentiated genes of clades in the core genome phylogenetic tree were identified using mafft (version 7.429). Then, those differentiated genes were defined as clade-specific genes and submitted to gene ontology (GO) term enrichment analysis by GOEAST (version 1.2.0)^7^.

### Statistical Analysis

Statistical analysis was performed using SAS Statistical Software (version 9.4). The Chi-square test (χ^2^) or Fisher’s exact test (two-tailed) was performed to compare data [including virulence factors, plasmids replicon types, and antibiotic resistance genes (ARGs)] of two groups. For each comparison, only a *p*-value < 0.05 was statistically significant.

## Results

### Molecular Characteristics of *Escherichia coli* ST1193

A total of 76 *E. coli* ST1193 genome sequences were included in this study. All of them were collected from humans from Fuzhou, China and non-China regions with a broad range of years. All *E. coli* ST1193 possessed *fimH64, filCH5*, and *fumC14* allele. There was 94.7% (72/76) of *E. coli* ST1193 classified as O-type O75. The remaining four isolates could not be typed.

Two types of capsule variants (K1 and K5) were found in this study. Among them, seven (9.33%) *E. coli* ST1193 possessed K5 capsular genotype, while 68 (90.67%) isolates possessed K1 capsular genotype. One isolate possessed neither K1 capsular genotype nor K5 capsular genotype. There was 16.67% (4/24) of non-Chinese *E. coli* ST1193 that possessed K5 capsular and the percentage was higher than that in isolates collected from Fuzhou, China (5.88%, 3/51).

**FIGURE 1:**
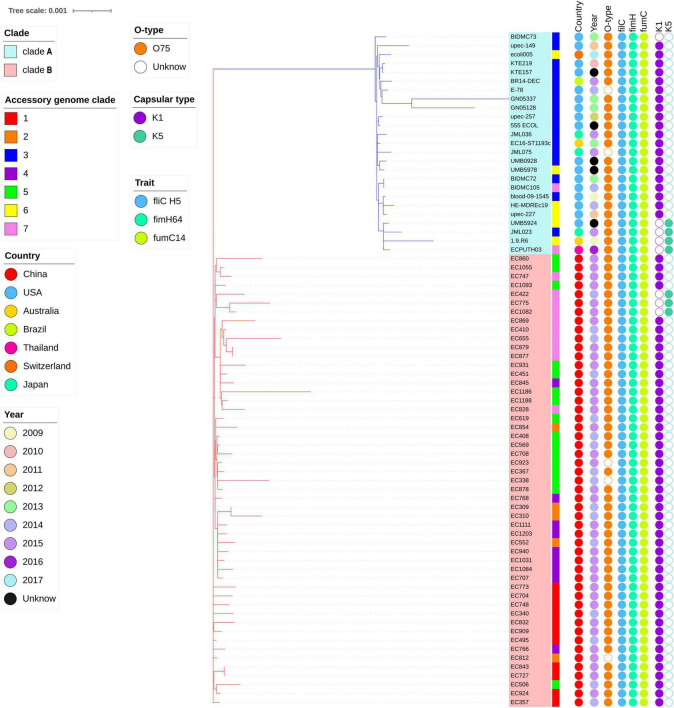
Phylogenetic tree of *E. coli* ST1193 based on single-copy gene in core genome. The phylogram was built from 3,454 single-copy core-genes using maximum likelihood (ML). Branch support was performed by Whelan-and-Goldman 2001 model and 1,000 bootstrap replicates. Taxon labels for clades A and B are colored blue and red, respectively. The accessory genome clades information is shown as colored strips surrounding the phylogram. Metadata are represented as circles as follows: year and geographical region in assorted colors as depicted in the legend. Allelic profiling information is shown as colored circles surrounding the phylogram (from left to right) for the O-type, *fliC*, *fimH*, *fumC*, K1, and K5 capsular genes.

The highly likely CRISPR arrays were not detected in all of *E. coli* ST1193 in this study. The results of *fimH* and *fumC* alleles, capsule variants, and O-types in *E. coli* ST1193 and the metadata information of each isolate are shown in Figure 1.

### Phylogenomic Analysis of *Escherichia coli* ST1193

**FIGURE 2:**
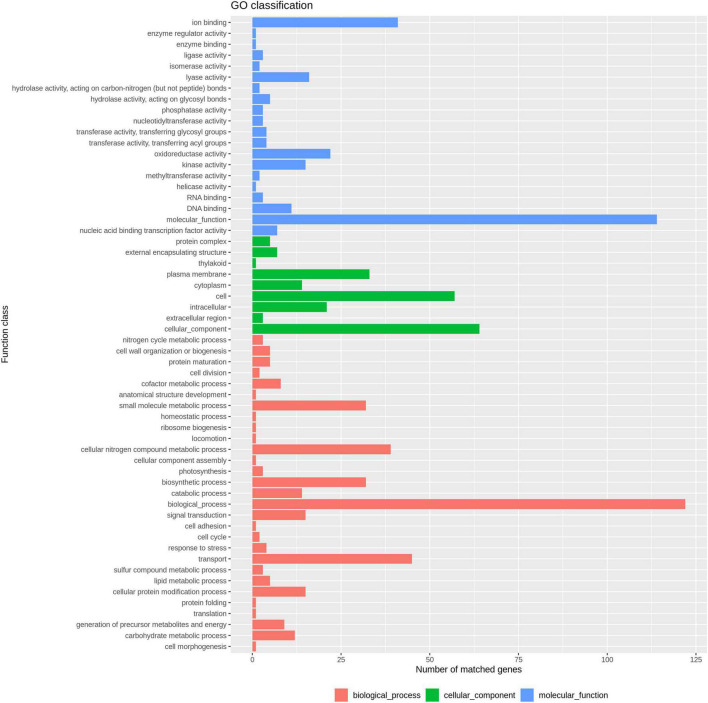
GO enrichment analysis result of 150 clade-specific genes.

In total, 3,454 single-copy core-genes were detected in the alignment. The core-genome alignments were applied for phylogenetic tree reconstruction using maximum likelihood estimation (Figure 1). All *E. coli* ST1193 were divided into two phylogenetic clades (clade A and B), with each supported by 100% bootstrapping. Clade A included 25 non-Chinese *E. coli* ST1193, and clade B contained all isolates from Fuzhou, China, respectively. Meanwhile, the isolates from clade B were more diverse than the entire worldwide collection. We further explored the difference of distinct clades in the core genome phylogenetic tree. The results of comparative genomics indicated that Indels were identified in a total of 150 clade-specific genes in our study (Supplementary Table 2). Based on the presence of these indels in particular genes, 76 *E. coli* ST1193 could be clearly clustered into two clades by geographical distribution. Besides, GO term enrichment analysis results showed that more than 70% of genes were enriched into the biological process and molecular function (Figure 2).

Furthermore, a total of 1,134 accessory genes were extracted to create an accessory genome matrix for all 76 *E. coli* ST1193 genomes. The accessory genome phylogenetic tree was obtained using maximum likelihood estimation (Supplementary Figure 1), resulting in seven distinct clades (clade 1–7). We mapped accessory genome clusters onto the core genome phylogeny (Figure 1). The results showed a high degree of correlation between accessory genome clusters (clades 3 and 6) and core genome phylogeny in clade A. Meanwhile, multiple accessory genome clusters were distributed throughout core genome phylogeny clade B that revealed the 51 *E. coli* ST1193 from Fuzhou, China with high genetic diversity.

### Virulence Factors

**FIGURE 3:**
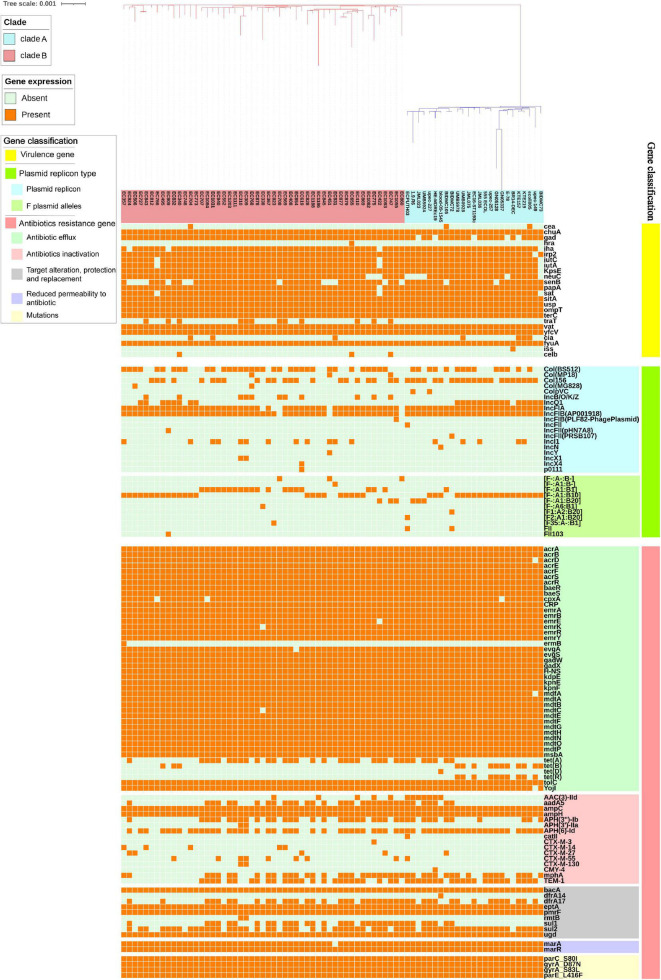
The virulence factors, plasmid replicon types, and antibiotics resistance genes in *E. coli* ST1193 genome. Heat map of 76 *E. coli* ST1193 genomes (51 isolates from Fuzhou, China and 25 isolates from non-China regions) showing the presence and absence of 92 antibiotic resistance genes (belonging to different categories, including antibiotic efflux, antibiotic inactivation, target alteration, protection and replacement, and reduced permeability to antibiotics) and four mutations in housekeeping genes; 24 virulence factors; 19 plasmid type and 11 F plasmid alleles. Gene names are represented on the Y axis and the *E. coli* ST1193 genomes are listed on the X axis.

The distribution of VFs differed among isolates (Figure 3). Some VFs were identified more than 70% among *E. coli* ST1193, such as outer membrane hemin receptor gene *chuA* (76/76, 100%), siderophore receptor gene *fyuA* (76/76, 100%), fimbrial protein gene *yfcV* (76/76, 100%), vacuolating autotransporter gene *vat* (76/76, 100%), asecreted autotransporter gene *sat* (72/76, 94.7%), IrgA homolog adhesin gene *iha* (71/76, 93.4%), glutamate decarboxylase gene *gad* (70/76, 92.1%), and plasmid-carried enterotoxin gene *senB* (58/76, 76.3%). These VFs mentioned above could be detected across all geographical regions.

**FIGURE 4:**
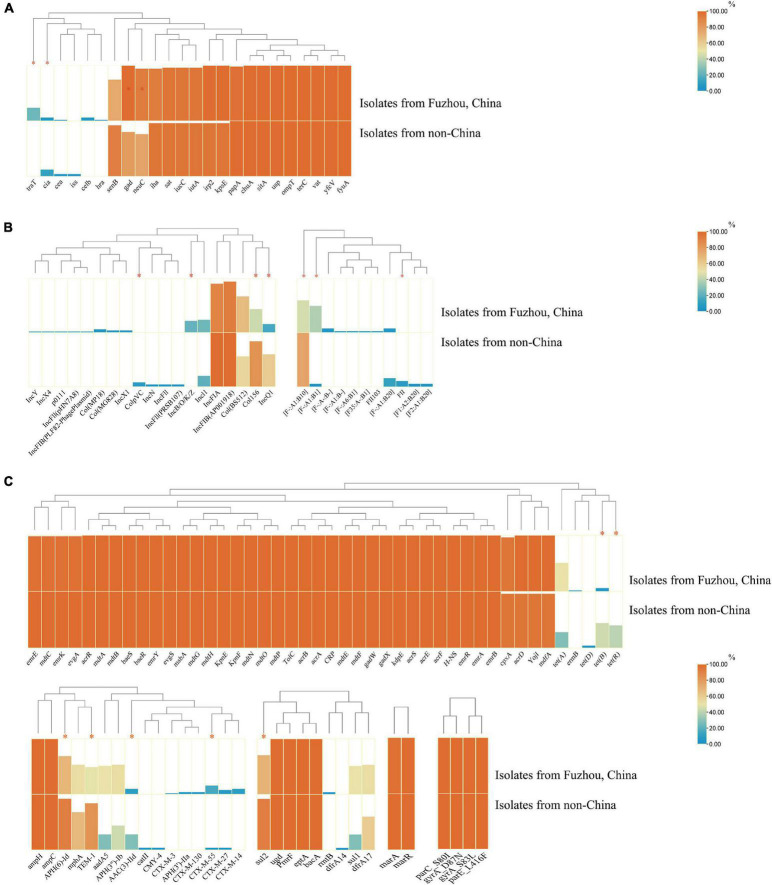
The comparison of virulence factors **(A)**, plasmid replicon types **(B)**, and antibiotics resistance genes **(C)** between isolates from non-China region (clade A) and isolates from Fuzhou, China (clade B). The comparison data were categorical variables and the Chi-square test (χ^2^) or Fisher’s exact test (two-tailed) were performed for data comparison of two groups. Only *p* < 0.05 was statistically significant. *p < 0.05.

Notably, there were significant differences in VFs between clade A (non-Chinese *E. coli* ST1193 isolates) and clade B (isolates from Fuzhou, China), including *cia*, *neuC*, *gad*, and *traT* (Figure 4A, *p* < 0.05). However, those differences were slight compared to the entire VFs profiles.

### Plasmids Replicon Types

Overall, we found that almost all isolates (75/76, 98.7%) carried at least one plasmid replicon type, covering 19 different known plasmid incompatibility types. Only one isolate (1.3%) contained no plasmid incompatibility types (Figure 3). Most *E. coli* ST1193 (73/76, 96.1%) contained two types of F-type replicons (IncFIA and IncFIB), while IncFII replicon was only detected in three strains. Plasmid multilocus sequence typing (MLST) based on F plasmid alleles was performed in this study. Our results revealed that IncFIA allele (A1) was highly prevalent across *E. coli* ST1193. IncFIB allele was diverse and four different alleles (B-, B1, B10 and B20) of IncFIB were identified. Col (BS512) and Col156 were highly prevalent among isolates from all geographical regions in this study.

Additionally, there were significant differences in plasmids replicon types carried between clade A (non-Chinese *E. coli* ST1193 isolates) and clade B (isolates from Fuzhou, China) (Figure 4B). IncQ1 was identified in 60.9% of non-Chinese *E. coli* ST1193, but found in only 39.1% of isolates from Fuzhou, China (*p* < 0.05). Col156 was detected in 84.0% of non-Chinese *E. coli* ST1193, but found in only 43.2% of isolates from Fuzhou, China (*p* < 0.05). IncB/O/K/Z was commonly found among *E. coli* ST1193 from Fuzhou, China (11/51, 21.6%), and undetected in non-Chinese isolates (0/25, 0%) (*p* < 0.05).

### Antibiotic Resistance Genes

The distribution of ARGs among *E. coli* ST1193 is shown in Figure 3. In this study, all ST1193 harbored the same four non-synonymous mutations in *parC* (S80I), *parE* (L416F), and *gyrA* (D87N and S83L) housekeeping genes (Figure 3). Mutations in those four housekeeping genes are known to confer fluoroquinolone resistance. Some antibiotic efflux genes and genes associated with reduced permeability to antibiotics were present in all 76 *E. coli* ST1193, such as *acrA*, *acrB*, *emrE*, and *mdtA*. Among *E. coli* ST1193, more than 50% isolates carried acquired antimicrobial resistance genes, including *bla*_*TEM–1B*_ (ampicillin resistance, 60.5%), *sul2* (sulfonamide resistance, 77.6%), *APH(6)-Id* (aminoglycoside resistance, 76.4%), *dfrA17* (trimethoprim resistance, 55.3%), and *mphA* (macrolide resistance, 57.9%). Notably, *bla*_*CTX–M–130*_ and *bla*_*CTX–M–14*_ were only detected in clade A (non-Chinese *E. coli* ST1193 isolates) and *tet(B)* and *tet(R)* were only identified in clade B (isolates from Fuzhou, China), respectively.

Furthermore, we observed significant differences in ARGs carried between clade A and clade B (Figure 4C, *p* < 0.05), including *bla*_*CTX–M–55*_, *bla*_*TEM–1*_, *sul2*, *tet(B)*, *tet(R)*, *APH(6)-Id*, and *AAC(3)-IId*.

### *bla*_*CTX–M–55*_ With Different Genetic Environments in *Escherichia coli* ST1193

**FIGURE 5:**
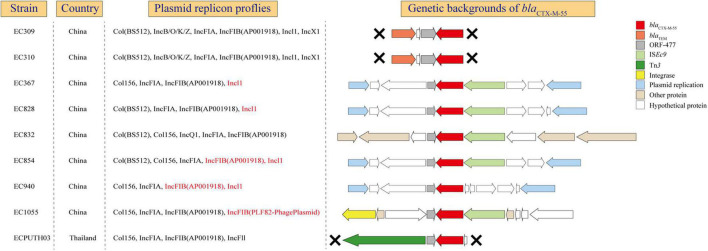
The genetic environments of *bla*_*CTX–M–55*_ in *E. coli* ST1193. X, gene contexts limited by the extent of the assembled region around the *bla*_*CTX–M–55*_. Red mark, *bla*_*CTX–M–55*_ located on the same contig with plasmid replicon.

*bla*_*CTX–M–55*_ was usually found in *E. coli* ST1193 as described previously and might play an important role in clonal expansion of ST1193 lineage, like *bla*_*CTX–M–15*_ in ST131 (Stoesser et al., 2016; Xia et al., 2017). This prompted us to further examine the genetic environment of this gene in ST1193. In our study, *bla*_*CTX–M–55*_ was found in nine isolates which all collected from Asia, including eight from Fuzhou, China and one from Thailand (Figure 5). From *bla*_*CTX–M–55*_ gene were present in plasmid-associated sequences in the contig assemblies, we could determine precise plasmid–AMR gene links for those isolates. In addition, we found that *bla*_*CTX–M–55*_ and IncI1 and/or FIB were identified in the same contig in 55.6% of isolates. In all nine isolates, *bla*_*CTX–M–55*_ was located on the downstream of a homologous tract of ORF477. Different mobile elements were detected on the downstream or the upstream of *bla*_*CTX–M–55*_, including *IS*Ec9, *Tn*3, and integrase gene. *bla*_*TEM–1*_ is located on the upstream of *bla*_*CTX–M–55*_ in two isolates from Fuzhou, China.

## Discussion

*Escherichia coli* ST1193 is a global pandemic strain and increasingly linked to various infections (Tchesnokova V. L. et al., 2019). The emergence and clinical significance of *E. coli* ST1193 suggested that fluoroquinolones-resistant is the signature feature of pandemic ST1193 clonal group (Wu et al., 2017; Tchesnokova V. et al., 2019). *E. coli* ST1193 spread rapidly worldwide during the last years (Tchesnokova V. L. et al., 2019). The increase and dissemination of *E. coli* ST1193 are of great concern. Although there has been increased research on *E. coli* ST1193 epidemiology and phylogenomics, the development of multidrug resistance and genomic diversification of ST1193 is not yet clear. In this study, we reported the whole genome sequences of 76 *E. coli* ST1193 from different countries. Combining analysis of core genome and accessory genome phylogeny, virulence factors, antibiotics resistance genes, and plasmid replicon profiles, we provide a significant insight for the important evolutionary characteristics and differences of *E. coli* ST1193, which supported its emergence as a dominant and successful MDR pathogen.

Based on genomic observations, we highlighted key characteristics of *E. coli* ST1193. Most of these strains were serotype O75. They all possessed *fimH*64, *filC*14, and *fumC*14 allele, which were similar to a previous study (Johnson T. J. et al., 2019). Furthermore, the CRISPR arrays were not found in any *E. coli* ST1193 in this study which was similar to a previous study (Al-Farsi et al., 2020).

WGS is an effective way to analyze the evolution and spread of bacterial strains (Knight et al., 2015; Meehan et al., 2019). Here, we combined core and accessory genomes to explore evolutionary patterns of separation within the population. A previous study demonstrated that *E. coli* ST1193 could be clustered to many distinct clades, but it could not verify geographical isolation sources and gain a confident conclusion regarding geographical clustering (Johnson T. J. et al., 2019). However, our study provides a new insight into the phylogeny of *E. coli* ST1193. There were 76 strains that could be clustered into two clades based on origin (Fuzhou, China and non-China) in a core genome phylogeny tree (Figure 1). Clade A contained 25 non-Chinese *E. coli* ST1193, and clade B included 51 *E. coli* ST1193 collected from Fuzhou, China. The results of core genome phylogenomic analysis of *E. coli* ST1193 indicates large differences between isolates from Fuzhou, China and non-China region, suggesting complex evolutionary processes underlying ST1193. A comparative genomics analysis was performed to demonstrate the reasons for the differences between two distance clades. Notably, indels were identified in a total of 150 clade-specific genes that enriched the biological process and molecular function in this study (Figure 2). Conserved indels of defined length and sequence were found at the same position in a given protein (or gene) in all members from one or more groups are useful to understand the evolutionary relationship of bacteria (Gupta, 2003). Our results indicated 150 indels were responsible for regional differences in *E. coli* ST1193 and were contributed to determine the relative branching orders of different clades in clade A (harboring 25 non-Chinese *E. coli* ST1193) and clade B (harboring 51 *E. coli* ST1193 from Fuzhou, China). A recent study highlighted that it is possible to provide a completely different perspective of the evolution of a well-defined or undefined bacterial lineage by combining core and accessory genome analysis (McNally et al., 2016). The accessory genome clusters of bacteria were due to the unique combinations of genes circulating in the accessory gene pool (Reuter et al., 2015). Therefore, we mapped the accessory genome clusters onto core genome phylogeny that showed a high degree of correlation between accessory genome profile and core genome phylogeny in clade A, and then multiple accessory genome clusters distributed throughout clade B (Figure 1 and Supplementary Figure 1). Our results indicated that *E. coli* ST1193 might contain multiple subtypes. Each of the subtypes arises due to the expansion of a successful clone following the emergence of a stable accessory gene profile. Interestingly, the results of core and accessory genome analysis demonstrated that geographical distributions might play a crucial role in the whole process throughout the phylogeny of *E. coli* ST1193. Furthermore, our study provided confident conclusions regarding geographical clustering for *E. coli* ST1193.

*Escherichia coli* ST1193 lineage exhibited a diversity of VFs profiles in this study. We found identical VFs profiles (*vat*, *fyuA*, *iutA*, *iha*, *gad*, *senB*, and *chuA*) among most ST1193 isolates (Figure 3). Those VFs were commonly prevalent in *E. coli* ST1193, consistent with a previous study (Valenza et al., 2019). Among those VFs, *senB* is a plasmid-carried enterotoxin gene (often detected in F-type plasmid) associated with enhancing bladder colonization and invasion (Cusumano et al., 2010). In addition, F-type plasmid replicons were most common in *E. coli* ST1193 in this study, including 100% in non-Chinese isolates and 94.2% in isolates from Fuzhou, China, respectively. Given this virulence factor’s presence in many successful ExPEC clones and the high prevalence of F-type plasmid in *E. coli* ST1193, it might be vital for ST1193’s epidemiologic success (Cusumano et al., 2010). Furthermore, we found significant differences were found in VFs (*neuC*, *gad*, and *traT*) between two clades (Figure 4A, *p* < 0.05). Among them, *traT* was related to the serum resistance of *E. coli* (Daga et al., 2019). *neuC* was the prerequisite VF in bloodstream infections (Čurová et al., 2020). *gad* participated in acid resistance in *E. coli* (Giangrossi et al., 2005). Based on the above-mentioned evidence, we suspect that *E. coli* ST1193 from Fuzhou, China might process better survivability than non-Chinese isolates. Nonetheless, more experimental evidence is needed to support this conclusion because there is a similar abundance of other VFs between the two groups.

*Escherichia coli* ST1193 lineage exhibited a diversity of antibiotics resistance gene profiles. In this study, all *E. coli* ST1193 isolates harbored the same four non-synonymous mutations in the target genes—two in *gyrA* (D87N and S83L), one in *parC* (S80I), and one in *parE* (L416F) and those mutations confer fluoroquinolone resistance (Figure 3). Our results supported the notion that the mutant variants of *gyrA* and *parC* were acquired not by a typical gradual, stepwise evolution but all at once (Tchesnokova V. et al., 2019). Notably, *E. coli* ST1193 from Fuzhou, China and non-Chinese isolates contained different ESBLs genes, which might be related to the epidemiology of those ESBLs genes and medication habits of different regions. Interestingly, *tet(B)* and *tet(R)* showed a higher prevalence in non-Chinese isolates in our study (*p* < 0.05). *tet(B)* [or *tet(R)*] is a type of tetracycline resistance genes, usually found in *E. coli* isolated from animals and environment (Seyfried et al., 2010; Lee et al., 2021). The difference prevalence of *tet(B)* [or *tet(R)*] between *E. coli* ST1193 from Fuzhou, China and non-Chinese ST1193 indicated that there might be a probable foodborne pathway to transmit *tet(R)* [or *tet(B)*] to humans *via* the food chain in a specified area. Additionally, the highly prevalent IncQ1 plasmid was observed within non-Chinese *E. coli* ST1193 (clade A) (*p* < 0.05). IncQ1 plasmid played a crucial role in the spread of tetracycline resistance, which might explain the high prevalence of *tet* genes in this study (Sun et al., 2019; Dungan and Bjorneberg, 2020). Mobile genetic elements could mediate independent microevolution of bacteria, such as ARGs and plasmids (Wu et al., 2019). Therefore, high dissemination of *tet* gene and IncQ1 within non-Chinese *E. coli* ST1193 isolates might participate in the microevolution of this lineage in a specified area.

A previous study suggested that the wide distribution of *bla*_*CTX–M*_ variants is mediated by clonal expansion of CTX-M containing *E. coli* and its global dissemination (Stoesser et al., 2016). Our study showed the persistence of particular *bla*_*CTX–M*_ variants (*bla*_*CTX–M–55*_) in *E. coli* ST1193 within specific geographic regions, such as Asia. *bla*_*CTX–M–55*_ was highly prevalent among *E. coli* isolates in China (Xia et al., 2017). In addition, the spread of this gene in China was mainly attributed to the clonal dissemination of *E. coli* ST1193 (Xia et al., 2017). Thus, we further explored the potential mechanisms of this gene at genetic levels (gene, genetic environments, and plasmid) (Figure 5). Our results showed that *bla*_*CTX–M–55*_ is located on IncI1 or IncFIB plasmids in most strains (55.6%), which was similar to a previous study from China (Xia et al., 2017). Meanwhile, the flanking contexts of *bla*_*CTX–M–55*_ in those strains were diverse, indicating that some different mechanisms (such as horizontal gene transfer) might play a significant role in microevolution in ST1193 lineage. The diverse genetic environments of *bla*_*CTX–M–55*_ might contribute to the global dissemination of this sequence type within specific geographic regions. As described previously, the almost ubiquitous presence of *bla*_*CTX–M–15*_ within ST131 H30Rx is most striking and strongly associated with the presence of an IncFII_AY458016-like replicon (Stoesser et al., 2016). Therefore, further investigation should be performed to determine whether *bla*_*CTX–M–55*_ within *E. coli* ST1193 and its associated replicon (IncI1 and/or IncFIB) contribute to subcluster and spread across geographic regions, like ST131 H30Rx.

There are several limitations to this study. First, since Chinese *E. coli* ST1193 were collected during a relatively short period of time, we did not elucidate the trends across years. Second, the short-read sequencing (Illumina NovaSeq platform) and limited transformant sequencing could not fully assess the flanking regions and plasmid structures across the entire data set. Third, the Chinese strains were only isolated from a single center (Fuzhou, China) and further study needs to increase the sample size, including new strains from other provinces of China. Meanwhile, the number of genomes from the rest of the world was very small. More strains from other countries should be included in further analyses to make the results of comparative genomics analysis more representatives in the future.

## Conclusion

Our study provides an important insight into the phylogenetic relationship of fluoroquinolones-resistant *E. coli* ST1193. Our results reveal that the distinct evolutionary trajectories of the spread of *E. coli* ST1193 in Fuzhou, China and non-China regions and the *E. coli* ST1193 isolates from Fuzhou, China seemed more diverse than the entire worldwide collection. That different phylogenetic relationship supports both global transmission and localized lineage expansion of this lineage following specific introductions into a geographic locality. Our results were a good pilotage analysis and could provide a start point for further extended analysis. Strategic and continued surveillance should be conducted to prevent the infections caused by *E. coli* ST1193.

## Data Availability Statement

The genome sequences of 51 *E. coli* ST1193 were deposited in GenBank under BioProject PRJNA670319 (https://www.ncbi.nlm.nih.gov/bioproject/?term=PRJNA670319).

## Ethics Statement

All procedures of this study involving clinical isolates and their associated metadata information were reviewed and approved by the Medical Ethics Committee of Fujian Medical University Union Hospital (2020KY0121).

## Author Contributions

JH, SCZ, SYZ, and QZ performed the experiments. JH drafted the manuscript. QZ and ZZ analyzed the data. YC, HQ, and MC supervised the study. BL designed the study and revised the manuscript critically for important intellectual content. All authors read and approved the final manuscript and contributed to manuscript revision, read, and approved the submitted version.

## Conflict of Interest

The authors declare that the research was conducted in the absence of any commercial or financial relationships that could be construed as a potential conflict of interest.

## Publisher’s Note

All claims expressed in this article are solely those of the authors and do not necessarily represent those of their affiliated organizations, or those of the publisher, the editors and the reviewers. Any product that may be evaluated in this article, or claim that may be made by its manufacturer, is not guaranteed or endorsed by the publisher.

## Footnotes

1
https://www.bioinformatics.babraham.ac.uk/projects/fastqc/

2
http://enterobase.warwick.ac.uk/

3
https://www.ncbi.nlm.nih.gov/

4
https://cge.cbs.dtu.dk/services/

5
https://card.mcmaster.ca/analyze/rgi

6
https://crisprcas.i2bc.paris-saclay.fr/CrisprCasFinder/Index

7
http://omicslab.genetics.ac.cn/GOEAST/index.php
