# Supplementary material for: Phylogenetic Analysis Reveals Distinct Evolutionary Trajectories of the Fluoroquinolones-Resistant Escherichia coli ST1193 From Fuzhou, China
Source: Front Microbiol. 2021 Nov 5;12:746995. doi: 10.3389/fmicb.2021.746995 (PMC8602892; doi:10.3389/fmicb.2021.746995)

**Supplemental Material**

**Phylogenetic analysis reveals distinct evolutionary trajectories of the fluoroquinolones-resistant *Escherichia coli* ST1193 from Fuzhou, China**

Jiangqing Huang^a^, Zhichang Zhao^b^, Qianwen Zhang^a^, Shengcen Zhang^a^, Shuyu Zhang^c^, Min Chen^c^, Hongqiang Qiu^b^,Yingping Cao^a,*^,Bin Li^a, *^

*^a^ Department of Clinical Laboratory,* *Fujian Medical University Union Hospital, Fuzhou, Fujian, 350001, China*

*^b^ Department of Pharmacy, Fujian Medical University Union Hospital, Fuzhou, Fujian, 350001, China*

*^c^ Department of Laboratory Medicine,* *Fujian Medical University, Fuzhou, Fujian, 350001, China*

***Correspondence**

Prof. Yingping Cao

*Department of Clinical Laboratory, Fujian Medical University Union Hospital, 29 Xinquan Rd., Fuzhou, Fujian, 350001. China;*

E-mail: caoyingping@aliyun.com

Prof. Bin Li

*Department of Clinical Laboratory, Fujian Medical University Union Hospital, 29 Xinquan Rd., Fuzhou, Fujian, 350001. China;*

E-mail:[*leonlee307@hotmail.com*](mailto:leonlee307@hotmail.com)

Table

Table S1. The Bioproject numbers and Biosample numbers of strains used in this study

| Number | Strain name | Bioject |
| --- | --- | --- |
| 1 | 1.9.R6 | PRJNA335932 |
| 2 | BR14-DEC | PRJNA511007 |
| 3 | EC16-ST1193c | PRJNA480723 |
| 4 | ECPUTH03 | PRJNA389557 |
| 5 | E-78 | PRJNA316969 |
| 6 | UMB0928 |  |
| 7 | UMB5978 |  |
| 8 | UMB5924 |  |
| 9 | GN05128 | PRJNA290784 |
| 10 | GN05337 |  |
| 11 | BIDMC105 | PRJNA271899 |
| 12 | 555_ECOL | PRJNA267549 |
| 13 | blood-09-1545 | PRJNA248737 |
| 14 | upec-227 |  |
| 15 | upec-257 |  |
| 16 | upec-49 |  |
| 17 | BIDMC73 | PRJNA234161 |
| 18 | BIDMC72 | PRJNA234160 |
| 19 | KTE219 | PRJNA163451 |
| 20 | KTE157 | PRJNA157707 |
| 21 | ecoli005 | PRJEB28660 |
| 22 | JML023 | PRJDB5579 |
| 23 | JML036 |  |
| 24 | JML075 |  |
| 25 | HE-MDREc19 | PRJDB4868 |

Table S2. The Bioproject numbers and Biosample numbers of strains used in this study

| Number | Genbank accession number | Gene annotation |
| --- | --- | --- |
| 1 | WP_077572252.1 | long-chain-fatty-acid--CoA ligase FadD |
| 2 | WP_001055794.1 | carboxy terminal-processing peptidase |
| 3 | WP_001298452.1 | lipid-binding membrane homeostasis protein YebT |
| 4 | WP_001538877.1 | chemotaxis protein CheA |
| 5 | WP_001773944.1 | cellulose biosynthesis regulator YedQ |
| 6 | WP_001773946.1 | site-specific integrase |
| 7 | WP_024193506.1 | tyrosine-type recombinase/integrase |
| 8 | WP_001286281.1 | yersiniabactin-associated zinc MFS transporter YbtX |
| 9 | WP_001304269.1 | yersiniabactin ABC transporter ATP-binding/permease protein YbtQ |
| 10 | WP_015912519.1 | toxic metabolite efflux MATE transporter YeeO |
| 11 | WP_000880178.1 | imidazole glycerol phosphate synthase subunit HisF |
| 12 | WP_001538929.1 | putative colanic acid polymerase WcaD |
| 13 | WP_032139937.1 | protein-serine/threonine phosphatase PphC |
| 14 | WP_001538960.1 | fimbrial biogenesis outer membrane usher protein |
| 15 | WP_001538973.1 | VWA domain-containing protein |
| 16 | WP_000876057.1 | two-component system sensor histidine kinase RcsC |
| 17 | WP_001774000.1 | glycerol-3-phosphate dehydrogenase subunit GlpB |
| 18 | WP_001539172.1 | M28 family peptidase |
| 19 | WP_000247881.1 | NADH-quinone oxidoreductase subunit C/D |
| 20 | WP_000062997.1 | NADH-quinone oxidoreductase subunit NuoA |
| 21 | WP_001335436.1 | LacI family DNA-binding transcriptional regulator |
| 22 | WP_000544377.1 | two-component system sensor histidine kinase YpdA |
| 23 | WP_000931396.1 | galactose-1-epimerase |
| 24 | WP_001538484.1 | malonyl-ACP O-methyltransferase BioC |
| 25 | WP_001538488.1 | ABC transporter ATP-binding protein |
| 26 | WP_100228301.1 | TMAO reductase system sensor histidine kinase/response regulator TorS |
| 27 | WP_001337722.1 | pyrimidine utilization protein D |
| 28 | WP_001538560.1 | ribonuclease E |
| 29 | WP_001116602.1 | thiamine kinase |
| 30 | WP_001043458.1 | glycine zipper 2TM domain-containing protein |
| 31 | WP_001294595.1 | methionine ABC transporter permease MetI |
| 32 | WP_001540727.1 | outer membrane usher protein |
| 33 | WP_001540723.1 | fimbrial-like protein YadL |
| 34 | WP_001540700.1 | protein transport protein HofC |
| 35 | WP_000785121.1 | UDP-N-acetylmuramoyl-L-alanyl-D-glutamate--2,6-diaminopimelate ligase |
| 36 | WP_001774153.1 | acetolactate synthase 3 large subunit |
| 37 | WP_001540677.1 | thiamine/thiamine pyrophosphate ABC transporter permease ThiP |
| 38 | WP_001540674.1 | thiamine ABC transporter ATP-binding protein ThiQ |
| 39 | WP_001088373.1 | YjjW family glycine radical enzyme activase |
| 40 | WP_001314410.1 | threonine/serine exporter ThrE family protein |
| 41 | WP_000538188.1 | threonine/serine exporter |
| 42 | WP_001701073.1 | peptide chain release factor 2 |
| 43 | WP_001539546.1 | urate/proton symporter UacT |
| 44 | WP_000004599.1 | lysophospholipid transporter LplT |
| 45 | WP_001539522.1 | prepilin peptidase-dependent protein |
| 46 | WP_001276487.1 | prepilin-type N-terminal cleavage/methylation domain-containing protein |
| 47 | WP_001539517.1 | exodeoxyribonuclease V subunit beta |
| 48 | WP_001539510.1 | type VI secretion system baseplate subunit TssG |
| 49 | WP_001556810.1 | hypothetical protein |
| 50 | WP_001539494.1 | hypothetical protein |
| 51 | WP_001045530.1 | 23S rRNA (cytidine(2498)-2'-O)-methyltransferase RlmM |
| 52 | WP_001539470.1 | YgcG family protein |
| 53 | WP_032143646.1 | sucrose-6-phosphate hydrolase |
| 54 | WP_000956642.1 | ATPase RavA stimulator ViaA |
| 55 | WP_001318143.1 | PTS beta-glucoside transporter subunit IIABC |
| 56 | WP_001113432.1 | DUF202 domain-containing protein |
| 57 | WP_001314234.1 | PRD domain-containing protein |
| 58 | WP_000360417.1 | lipopolysaccharide core heptosyltransferase RfaQ |
| 59 | WP_001304976.1 | O-antigen ligase family protein |
| 60 | WP_000893156.1 | L-ribulose-5-phosphate 4-epimerase |
| 61 | WP_001297957.1 | protein bax |
| 62 | WP_001540040.1 | biotin sulfoxide reductase |
| 63 | WP_074159041.1 | cellulose biosynthesis protein BcsE |
| 64 | WP_001540016.1 | cellulase |
| 65 | WP_001266324.1 | biofilm formation regulator HmsP |
| 66 | WP_000723717.1 | NADH-dependent methylglyoxal reductase |
| 67 | WP_001557005.1 | ABC transporter permease |
| 68 | WP_001241562.1 | cell division activator CedA |
| 69 | WP_001538789.1 | electron transport complex subunit RsxC |
| 70 | WP_000890295.1 | glycyl radical protein |
| 71 | WP_001216482.1 | two-component system sensor histidine kinase DcuS |
| 72 | WP_000507111.1 | D-allose ABC transporter permease |
| 73 | WP_011076734.1 | formate dehydrogenase H subunit alpha, selenocysteine-containing |
| 74 | WP_000956822.1 | Na/Pi cotransporter family protein |
| 75 | WP_001269824.1 | molecular chaperone |
| 76 | WP_000096091.1 | YqjK-like family protein |
| 77 | WP_001295548.1 | PTS mannose/fructose/sorbose transporter family subunit IID |
| 78 | WP_000934823.1 | protein translocase subunit SecD |
| 79 | WP_000154044.1 | phosphatidylglycerophosphatase A |
| 80 | WP_001538382.1 | SEL1-like repeat protein |
| 81 | WP_001239434.1 | cytochrome o ubiquinol oxidase subunit II |
| 82 | WP_001538391.1 | SmdB family multidrug efflux ABC transporter permease/ATP-binding protein |
| 83 | WP_001295323.1 | SmdB family multidrug efflux ABC transporter permease/ATP-binding protein |
| 84 | WP_023151267.1 | iron export ABC transporter permease subunit FetB |
| 85 | WP_001386103.1 | multifunctional acyl-CoA thioesterase I/protease I/lysophospholipase L1 |
| 86 | WP_001774064.1 | phosphoglycolate phosphatase |
| 87 | WP_000816006.1 | DNA uptake porin HofQ |
| 88 | WP_001350442.1 | DNA utilization protein GntX |
| 89 | WP_001539963.1 | molecular chaperone |
| 90 | WP_001557129.1 | fimbrial biogenesis usher protein |
| 91 | WP_001098636.1 | dipeptide/tripeptide permease DtpB |
| 92 | WP_024193510.1 | MgtC/SapB family protein |
| 93 | WP_001538419.1 | bacteriophage adsorption protein NfrA |
| 94 | WP_001538421.1 | Cu(+)/Ag(+) sensor histidine kinase CusS |
| 95 | WP_001538436.1 | hypothetical protein |
| 96 | WP_001043146.1 | carbon starvation protein CstA |
| 97 | WP_000126500.1 | two-component response regulator DpiA |
| 98 | WP_001538463.1 | D-threonate 4-phosphate dehydrogenase |
| 99 | WP_001538464.1 | two-component system sensor histidine kinase KdpD |
| 100 | WP_000254365.1 | succinate dehydrogenase membrane anchor subunit |
| 101 | WP_001443189.1 | acyltransferase |
| 102 | WP_001540266.1 | STM4012 family radical SAM protein |
| 103 | WP_012602895.1 | formate dehydrogenase-N subunit alpha |
| 104 | WP_000204136.1 | [formate-C-acetyltransferase]-activating enzyme |
| 105 | WP_000204136.1 | [formate-C-acetyltransferase]-activating enzyme |
| 106 | WP_000859934.1 | cyclic di-GMP phosphodiesterase |
| 107 | WP_001250202.1 | peptide ABC transporter substrate-binding protein SapA |
| 108 | WP_001538707.1 | carbohydrate ABC transporter permease |
| 109 | WP_001538710.1 | Gfo/Idh/MocA family oxidoreductase |
| 110 | WP_071590417.1 | DHA2 family efflux MFS transporter permease subunit |
| 111 | WP_001350251.1 | YdbL family protein |
| 112 | WP_001350251.1 | YdbL family protein |
| 113 | WP_001540519.1 | tRNA (adenosine(37)-N6)-threonylcarbamoyltransferase complex ATPase subunit type 1 TsaE |
| 114 | WP_001299664.1 | PTS trehalose transporter subunit IIBC |
| 115 | WP_000584107.1 | LPS export ABC transporter permease LptF |
| 116 | WP_000074795.1 | transcriptional regulator NanR |
| 117 | WP_001539851.1 | AsmA2 domain-containing protein YhdP |
| 118 | WP_000123197.1 | ribonuclease G |
| 119 | WP_001774054.1 | sodium/pantothenate symporter |
| 120 | WP_001219652.1 | tRNA dihydrouridine synthase DusB |
| 121 | WP_001538300.1 | DUF4150 domain-containing protein |
| 122 | WP_000532690.1 | Ivy family C-type lysozyme inhibitor |
| 123 | WP_001538304.1 | flagellar biosynthesis protein FlhA |
| 124 | WP_001265646.1 | fimbrial adhesin EcpD |
| 125 | WP_001304867.1 | LysR family transcriptional regulator |
| 126 | WP_001539293.1 | cysteine synthase B |
| 127 | WP_000021034.1 | sulfate/thiosulfate ABC transporter ATP-binding protein CysA |
| 128 | WP_001539307.1 | ethanolamine utilization ethanol dehydrogenase EutG |
| 129 | WP_000733877.1 | ethanolamine utilization acetate kinase EutQ |
| 130 | WP_001297612.1 | two component system sensor histidine kinase QseE/GlrK |
| 131 | WP_001116472.1 | 2-dehydro-3-deoxy-6-phosphogalactonate aldolase |
| 132 | WP_000070491.1 | two-component system response regulator NarL |
| 133 | WP_001538643.1 | PTS-dependent dihydroxyacetone kinase operon transcriptional regulator DhaR |
| 134 | WP_000340216.1 | potassium/proton antiporter |
| 135 | WP_001266908.1 | D-amino acid dehydrogenase |
| 136 | WP_000969007.1 | diguanylate cyclase DgcN |
| 137 | WP_032140825.1 | HlyC/CorC family transporter |
| 138 | WP_001117834.1 | RnfH family protein |
| 139 | WP_000774978.1 | glycine betaine/L-proline ABC transporter permease ProW |
| 140 | WP_001058513.1 | type II secretion system minor pseudopilin GspK |
| 141 | WP_000610632.1 | type II secretion system minor pseudopilin GspJ |
| 142 | WP_001773915.1 | zinc-dependent metallopeptidase |
| 143 | WP_011478186.1 | formate dehydrogenase-N subunit alpha |
| 144 | WP_000045647.1 | formate dehydrogenase-N subunit gamma |
| 145 | WP_001538755.1 | glycoside hydrolase family 10 protein |
| 146 | WP_000644685.1 | murein transglycosylase D |
| 147 | WP_001300651.1 | DUF1481 domain-containing protein |
| 148 | WP_001540375.1 | thiazole biosynthesis adenylyltransferase ThiF |
| 149 | WP_001538615.1 | spermidine/putrescine ABC transporter permease PotB |
| 150 | WP_000829599.1 | DUF4150 domain-containing protein |

Figure

Figure legend

Figure S1. **Phylogenetic tree of *E. coli* ST1193 based on single-copy gene in accessory genome.**The phylogram was built from 1,134 single-copy accessory genes using maximum likelihood (ML).Branch support was performed by Whelan-And-Goldman 2001 model and 1000 bootstrap replicates. The accessory genome clades information is shown as colored stripssurrounding the phylogram.

FigureS1.


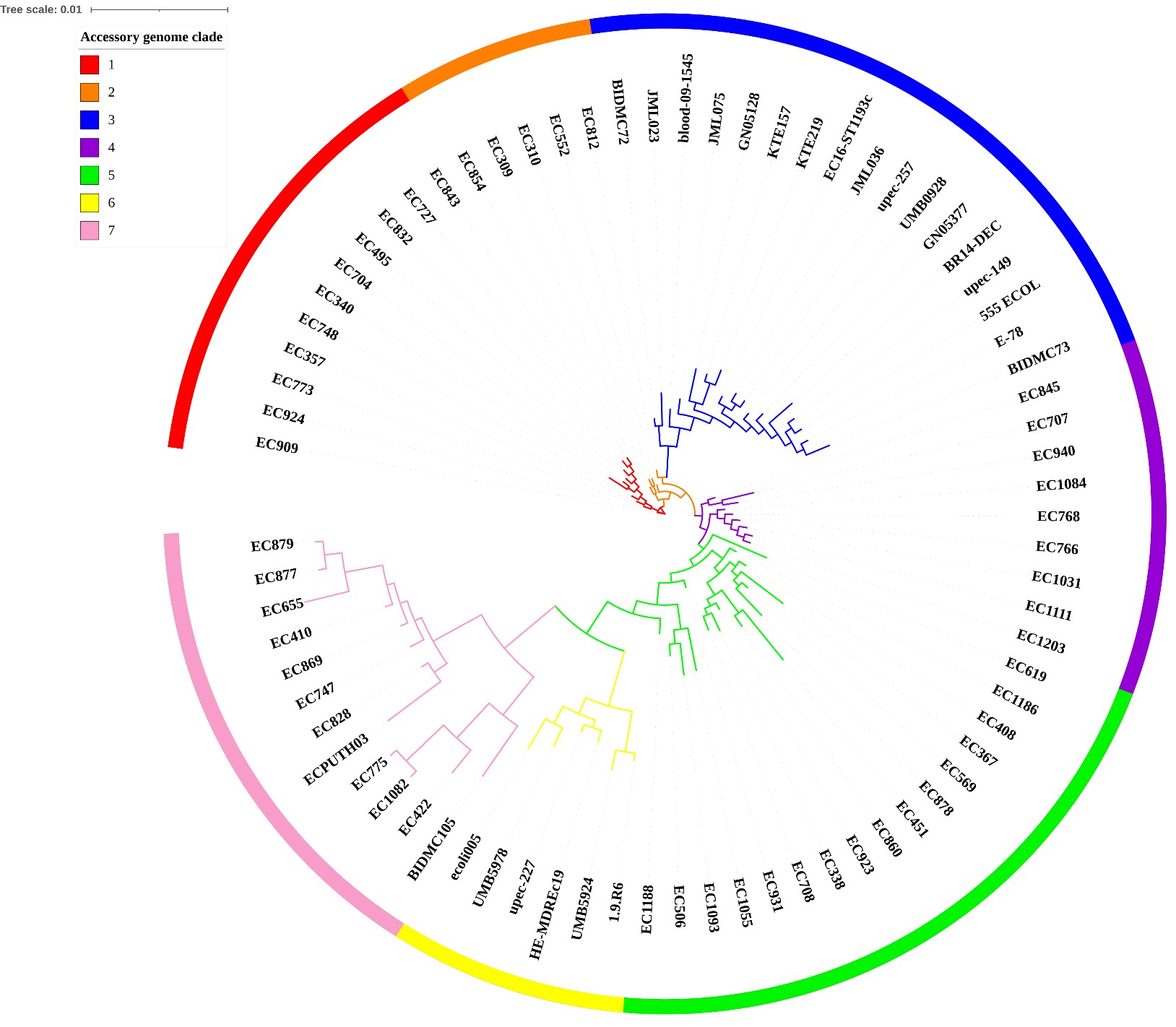

Supplement: Supplementary file 1 [file Data_Sheet_1.docx]
